# Post-infarction ventricular septal defect: triggered by Valsalva manoeuvre?

**DOI:** 10.5830/cvja-2010-011

**Published:** 2010-12

**Authors:** Baskurt Murat, Turhan Nihan, Kucukoglu Serdar, Hatemi Alican, Canikoglu Mustafa, Karadag Bilgehan

**Affiliations:** Department of Cardiology, Institute of Cardiology, Istanbul University, Haseki, Istanbul; Department of Cardiology, Institute of Cardiology, Istanbul University, Haseki, Istanbul; Department of Cardiology, Institute of Cardiology, Istanbul University, Haseki, Istanbul; Department of Cardiovascular Surgery, Institute of Cardiology, Istanbul University, Haseki, Istanbul; Department of Cardiovascular Surgery, Institute of Cardiology, Istanbul University, Haseki, Istanbul; Department of Cardiology, Cerrahpasa School of Medicine, Istanbul University, Cerrahpasa, Istanbul

**Keywords:** ventricular septal defect, post myocardial infarction complications

## Abstract

Post–infarction ventricular septal defect (VSD) is a fatal mechanical complication of myocardial infarction. Although the incidence has decreased to less than 1% after the extensive use of reperfusion strategies, post–infarction VSD still carries a high mortality risk. Management is controversial, whether to wait for surgery after a stabilisation period or to perform emergency surgery when diagnosed. We report on a case of post–infarction VSD that was detected with severe haemodynamic instability, beginning immediately after the patient’s Valsalva manoeuvre on the sixth day of a non–reperfused inferior myocardial infarction. In the early period, the post–infarction VSD was repaired via a trans–aneurysmal approach.

The incidence of post–infarction ventricular septal defect (VSD) was 1–3% in the pre–thrombolytic era, but it has declined to nearly 0.2% in the era of thrombolysis.^1^ The average time interval for a post–infarction VSD to occur is at five to six days if thrombolytic therapy is not used, and one day with thrombolytic administration.^2^ In–hospital mortality is still more than 90% with medical therapy and between 19 and 60% with a surgical approach.^3^ VSD complicating an inferior myocardial infarction (MI) has a poorer prognosis than a VSD complicating an anterior MI.^4^

We report on a case of post–infarction VSD in a patient who did not receive thrombolytic therapy because of late presentation. On the sixth day of the inferior MI, after a period of Valsalva manoeuvre, she developed sudden haemodynamic deterioration and symptoms and signs of acute heart failure. After the diagnosis of post–infarction VSD, we performed emergency surgery and repaired the defect by teflon strip with pladgeted prolene sutures via a trans–aneurysmal approach.

## Case report

A 62–year–old female patient was admitted to our hospital with typical enduring chest pain that had started two days earlier. Her past medical history was normal and she was a non–smoker. Her physical examination was normal. Surface electrocardiogram showed 2–mm ST elevation in leads D2, D3 and aVF with pathological Q waves and negative T waves, suggesting a sub–acute phase of inferior MI. Her biochemical markers of MI were higher than the upper limits of normal.

She was admitted to the coronary care unit with a diagnosis of sub–acute inferior MI and started on isosorbite mononitrate 60 mg/day (p.o.), metoprolol succinate 100 mg/day (p.o.), acetylsalicylic acid 300 mg/day (p.o.), clopidogrel 75 mg/day (p.o.) and enoxaparine 1 mg/kg, bid, (s.c.). Transthoracic echocardiography was performed in the first hour after admission to the coronary care unit on day one and showed akinesia in the basal septum and mid–basal inferior wall, with a calculated ejection fraction of 45%.

The patient was transferred to a room after an uncomplicated course of 36 hours. On the second day, at 08:00 her medical examination was normal. One hour later, after using the toilet and after several attempts at Valsalva manoeuvres, she felt sudden chest pain, dyspnoea and nausea. Her heart rate was 110/minute, blood pressure was 80/60 mmHg, and a new systolic murmur was recorded at the left sternal border with thrill and bilateral rales at the basal level of both lungs. Her surface electrocardiogram was unchanged. She was immediately transferred to the coronary care unit. This was the fourth day after admission to hospital.

**Fig. 1:**
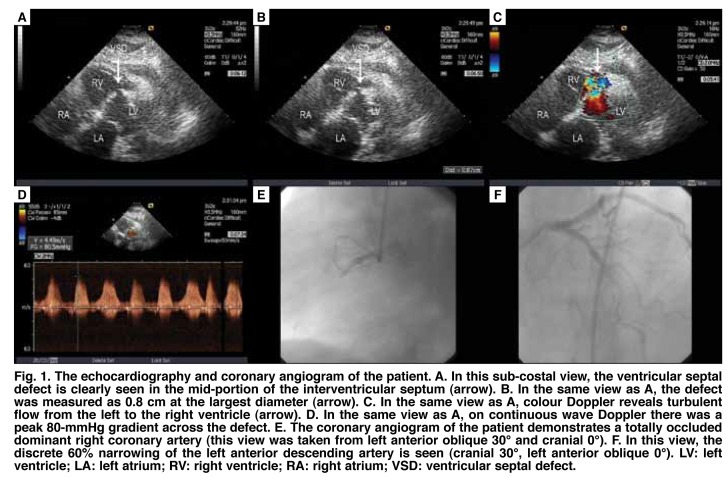
The echocardiography and coronary angiogram of the patient. A. In this sub–costal view, the ventricular septal defect is clearly seen in the mid–portion of the interventricular septum (arrow). B. In the same view as A, the defect was measured as 0.8 cm at the largest diameter (arrow). C. In the same view as A, colour Doppler reveals turbulent flow from the left to the right ventricle (arrow). D. In the same view as A, on continuous wave Doppler there was a peak 80–mmHg gradient across the defect. E. The coronary angiogram of the patient demonstrates a totally occluded dominant right coronary artery (this view was taken from left anterior oblique 30° and cranial 0°). F. In this view, the discrete 60% narrowing of the left anterior descending artery is seen (cranial 30°, left anterior oblique 0°). LV: left ventricle; LA: left atrium; RV: right ventricle; RA: right atrium; VSD: ventricular septal defect.

A second transthoracic echocardiography was performed and revealed a left ventricular infero–basal aneurysm, infero–mid and infero–septal akinesia and a ventricular septal defect in the basal mid–interventricular septum, creating a peak 80–mmHg gradient and mild pericardial effusion (Fig. 1a–d). Intravenous saline was started and after stabilising her haemodynamic status, a coronary angiogram was performed the following day. It showed total occlusion in the mid segment of a dominant right coronary artery and a discrete 60% narrowing in the mid–portion of the left anterior descending artery (Fig. 1e, f). She had recurrent hypotension and dyspnoea after catheterisation, and an intra–aortic balloon pump (IABP) was inserted. Early surgical repair was planned.

**Fig. 2:**
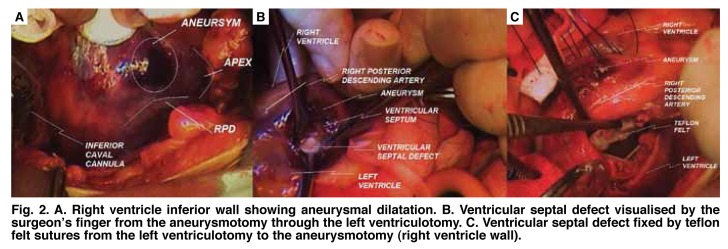
A. Right ventricle inferior wall showing aneurysmal dilatation. B. Ventricular septal defect visualised by the surgeon’s finger from the aneurysmotomy through the left ventriculotomy. C. Ventricular septal defect fixed by teflon felt sutures from the left ventriculotomy to the aneurysmotomy (right ventricle wall).

The patient underwent an operation two days after successful IABP insertion in the coronary care unit. The operation was performed with aorto–bicaval cannulation under extra–corporeal circulation (ECC). The heart was arrested by anterograde and retrograde blood cardioplegia. Exploration of the ventricular septum showed an inferior aneurysm in the right ventricular side approach (Fig. 2). Inspection showed no other aneurysm formation, necrosis or ischaemic region.

The ventricular septal defect that was near the apex of the septum was closed with the trans–aneurysmal approach. It was infixed to the left ventricular free wall to be repaired by teflon strip with pladgeted prolene sutures. The aneurysmotomy was closed in a buttress of strips of teflon felt and sutured with pladgeted prolene sutures (Fig. 2). After a right coronary artery bypass with a saphenous vein graft, anostomosis of the left internal mammary artery to the left anterior descending artery was performed.

There was no difficulty weaning the patient from the ECC with 2:1 IABP assistance in sinus rhythm. The postoperative course was uncomplicated. Early transthoracic echocardiography revealed no shunt. The patient was weaned from the IABP on the first postoperative day and inotropic drugs were reduced on the third postoperative day with stable haemodynamic parameters.

## Discussion

Post–infarction VSD was first shown by Latham in an autopsy in 1845, and clinical diagnostic criteria were first introduced by Sager in 1934.^5^ Reported studies defined risk factors for developing post–infarction VSD to be advanced age, female gender, hypertension, non–smoker, higher Killip score at admission, no known coronary heart disease and first MI.^6^,^7^

Post–infarction VSD is usually located in the anterior or apical portion of the ventricular septum (about 60% of cases) as a result of an anterior MI. Twenty to 40% of patients have a defect in the posterior portion of the ventricular septum as a result of an inferior MI.^2^ However, unexpected locations for ruptures also appear in the literature.^1^ VSD is usually associated with complete obstruction of a coronary artery.^6^

Symptoms are mostly chest pain, dyspnoea, and symptoms due to low output and cardiogenic shock.^2^ Almost all patients have a new harsh, loud, pansystolic murmur located at the left sternal border, spreading to the apex. A thrill usually accompanies this. Doppler echocardiography has 100% sensitivity and specificity for diagnosis.^1^ Transoesophageal echocardiography and right heart catheterisation are further techniques when transthoracic echocardiography is inconclusive but with a high clinical suspicion.^1^

In patients who do not receive thrombolytic therapy, coagulation necrosis begins within three to five days of infarction. Neutrophils penetrate into the necrotic zone. By triggering apoptosis, released lytic enzymes cause the necrotic myocardium to rupture.^1^ With the rupture of the septum, a shunt from the left to the right ventricle occurs, which increases volume load of the right ventricle and also pulmonary blood flow, left atrial and left ventricular volumes. The magnitude of VSD, pulmonary– systemic vascular resistance, Qp/Qs ratio and left–right ventricular functions determine the amount of shunt.^1^ If left ventricular systolic functions deteriorate, forward flow would decrease and compensatory vasoconstriction would raise systemic vascular resistance, leading to increased shunt flow.

Although the exact mechanism of rupture was not known, we presume that on the sixth day post MI, after our patient performed several forceful Valsalva manoeuvres, the thinned, necrotic septum was affected by the sudden volume and pressure changes created by the Valsalva manoeuvres. This may have caused the septum to rupture.

It has been shown that an approximately 20–mmHg fall in the systemic blood pressure occurs during early phase 2, which is followed by an abrupt rise in phase 4 of the Valsalva manoeuvre (about 43 mmHg) in a patient who is sitting and performing it.8 An overshoot of arteriolar vasoconstriction during late phase 2 and a 42% transient increase of the cardiac output above resting state are believed to be the reason for the blood pressure rise during phase 4.9 ACC/AHA practice guidelines recommend daily stool softeners in the hospital management of ST–elevation MI and our opinion is that this recommendation should be closely followed.^10^

Previously reported studies have shown that mortality is higher with: early VSD with surgery in the first 24 hours, inferior MI, the need for inotropic agents, cardiogenic shock, low mean pulmonary arterial pressure (PAP) and pulmonary capillary wedge pressure (PCWP), low cardiac index (CI < 1.75 ml/min/ m^2^), serious right ventricular dysfunction and low systolic blood pressure.4,11 If the right ventricle can handle the volume overload, the mean PAP and PCWP increases, as well as the survival rate.^11^

The first repair by Cooley et al. in 1957, a case of acquired ventricular septal defect, was accomplished using an approach through the right ventricle with incision of the right ventricular outflow tract.^12^ This approach, which was adapted from surgical techniques for closure of congenital ventricular septal defects, proved to be disadvantageous for many reasons. Subsequently, Heimbecker et al. introduced and others adopted a left–sided approach (left ventriculotomy) with incision through the area of infarction. Such an approach frequently incorporates infarctectomy and aneurysmectomy, together with repair of the septal rupture.^12^

Our choice was to attach the apical–septal VSD to the left ventricular free wall and repair with teflon strips and pladgeted prolene sutures. The aneurysmotomy was closed and buttressed with strips of teflon felt and sutured with pladgeted prolene sutures after RCA bypass with a saphenous vein. The left internal mammary artery to the left anterior descending anastomosis was performed at the last stage.

It was recently reported that a small or medium–sized postinfarction VSD could be treated successfully with a ventricular septal occluder. This intervention may be permanent or it may stabilise the patient and allow myocardial fibrosis, thus facilitating delayed subsequent surgical correction.^13^

## Conclusion

Post–infarction VSD is a serious mechanical complication of MI. Close monitoring of the non–reperfused patients is mandatory and a forceful Valsalva manoeuvre may facilitate the rupture of the myocardium, causing post–infarction VSD. Repair of postinfarction VSD can be accomplished with a trans–aneurysmal approach. The potential advantage of this technique is that an incision in the intact left ventricular myocardium is avoided.
